# Case Report: CTC1 mutations in a patient with diffuse hepatic and splenic hemangiomatosis complicated by Kasabach–Merritt syndrome

**DOI:** 10.3389/fonc.2023.1087790

**Published:** 2023-01-25

**Authors:** Xin He, Zi-Wen Guo, Xiao-Min Niu

**Affiliations:** Department of Hematology, Zhongshan People’s Hospital, Zhongshan, China

**Keywords:** diffuse hepatic hemangiomatosis, adult Kasabach-Merritt syndrome, *CTC1* gene, combination therapy, vascular disorder

## Abstract

Diffuse hemangiomatosis of the liver and spleen is rare. Currently, few studies are available on diffuse hepatic and splenic hemangiomatosis accompanied by Kasabach–Merritt syndrome (KMS). The conserved telomere maintenance component 1 (*CTC1*) gene contributes to telomere maintenance and replication by forming the telomeric capping complex. Herein, we report a case of diffuse hemangiomatosis in the liver and spleen accompanied by KMS in a 59-year-old woman who carried two novel heterozygous CTC1 variants: c.435+9A>C and c.3074C>T (p.Ala1025Val). Using next-generation sequencing, we detected mutations in the *CTC1* gene in our patient, who had chief complaints of fatigue and abdominal distension complicated by severe thrombocytopenia and consumptive coagulopathy. Clinical symptoms, laboratory tests, and imaging findings led to the diagnosis of diffuse hepatic and splenic hemangiomatosis accompanied by KMS. The patient was treated with prednisone, thalidomide, and sirolimus, and her general condition was ameliorated at the 4-month follow-up with improved platelet count and coagulation function. A *CTC1* gene mutation may be involved in the pathological process of vascular diseases. A combination treatment regimen of prednisone, thalidomide, and sirolimus may be effective for KMS.

## Introduction

Diffuse hemangiomatosis of the liver and spleen is an extremely rare disease in adults (1). Although most hepatic hemangiomas are benign, they can be fatal when accompanied by Kasabach–Merritt syndrome (KMS), which usually presents as thrombocytopenia, hemolytic anemia, and consumption coagulopathy (2). The conserved telomere maintenance component 1 (*CTC1*) gene contributes to telomere maintenance, replication, and genetic stability by forming the telomeric capping complex (3). *CTC1* gene mutations have been shown to cause cerebroretinal microangiopathy with calcifications and cysts (CRMCC), an autosomal recessive disorder, and dyskeratosis congenita (DC), a telomere biology disorder (4). Additionally, the *CTC1* gene has been found to be expressed in endothelial cells (5). *CTC1* gene mutations in diffuse hepatic hemangiomas accompanied by KMS remain unreported.

## Case presentation

**Figure 1:**
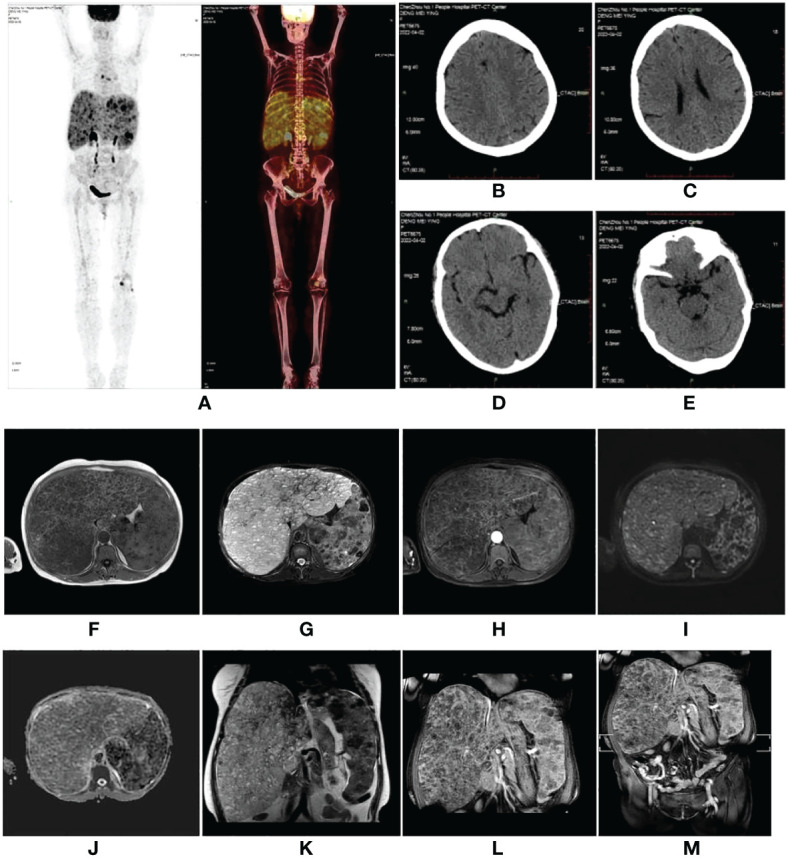
**(A–E)** Fluorodeoxyglucose positron emission tomography (FDG-PET) scan performed at the local hospital. The images showed diffuse hepatomegaly and splenomegaly with a slightly increased FDG uptake, without other abnormal FDG accumulation or intracranial lesions. **(F–M)** A magnetic resonance imaging scan of the abdomen revealed hepatosplenomegaly with countless nodules of various sizes involving the whole liver and spleen, which showed hypointensity on T1-weighted (T1W) images **(F)**, hyperintensity on T2-weighted (T2W) images **(G)**, and inhomogeneous enhancement in diffusion MRI **(I, H, L)**. Diffusion-weighted imaging (DWI) **(I)** showed a slightly higher signal intensity; however, the apparent diffusion coefficient (ADC) **(J)** value did not decrease. **(F)** T1W image. **(G)** Fat-saturated T2-weighted sequence (fsT2W). **(H)** The enhanced scan in the transverse view. **(I)** DWI. **(J)** ADC. **(K)** T2W image in the coronal view. **(L)** The enhanced scan in coronal view. **(M)** The enhanced scan of the whole abdomen in coronal view.

A 59-year-old woman who presented with abdominal distension, fatigue, and skin ecchymosis was referred to our hospital on 12 April 2022. From the medical history, we discovered that the patient experienced asthenia and abdominal distension 1 month previously, and jaundice and skin ecchymoses were exhibited 1 week later. She denied having any other medical or family history or a history of any drug use. Laboratory tests at the local hospital showed anemia, thrombocytopenia, prolonged prothrombin time, hypofibrinogenemia, and hyperbilirubinemia. A fluorodeoxyglucose positron emission tomography (FDG-PET) scan at the local hospital revealed diffuse hepatomegaly and splenomegaly with a slight increase in FDG uptake. Other metabolic abnormalities or intracranial lesions, such as leukoencephalopathy, parenchymal cysts, and intracranial calcification, were not observed (**Figure 1**). The patient’s condition worsened despite receiving a blood transfusion and expected treatment. Diffuse hepatomegaly, splenomegaly, and ecchymoses without cutaneous hemangioma or neurological disorders were detected during the physical examination. Further examinations, including blood tests and enhanced magnetic resonance imaging (MRI), were performed when the patient was referred to our hospital. Detailed patient characteristics are shown in **Table 1**.

**Table 1:** Clinical characteristics of the patient.

Patient sex/age (years)	Occupation	Symptoms	Physical examination	Medical or family history	Drug use history	Past interventions and outcomes	Mutation
**Female/59**	Farmer	Fatigue, abdominal distension	Hepatomegaly, splenomegaly, and ecchymoses	No	No	Blood transfusion, expectant treatment. The condition worsened.	CTC1: c.3074C>T (p.Ala1025Val); c.435+9A>C.

**Figure 2:**
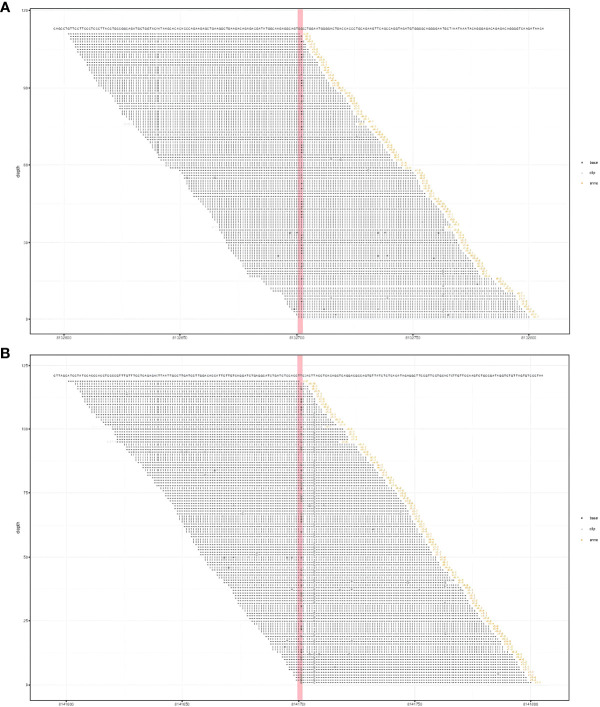
Results of whole exome sequencing showed compound heterozygous variants in the *CTC1* gene with a c.3074C>T (p.Ala1025Val) mutation (exon 19) **(A)** and a c.435+9A>C mutation (IVS3) **(B)**. **(A)** CTC1 NM_025099.5 exon 19 c.3074C>T (p.Ala1025Val). **(B)** CTC1 NM_025099.5 IVS3 c.435+9A>C.

**Figure 3:**
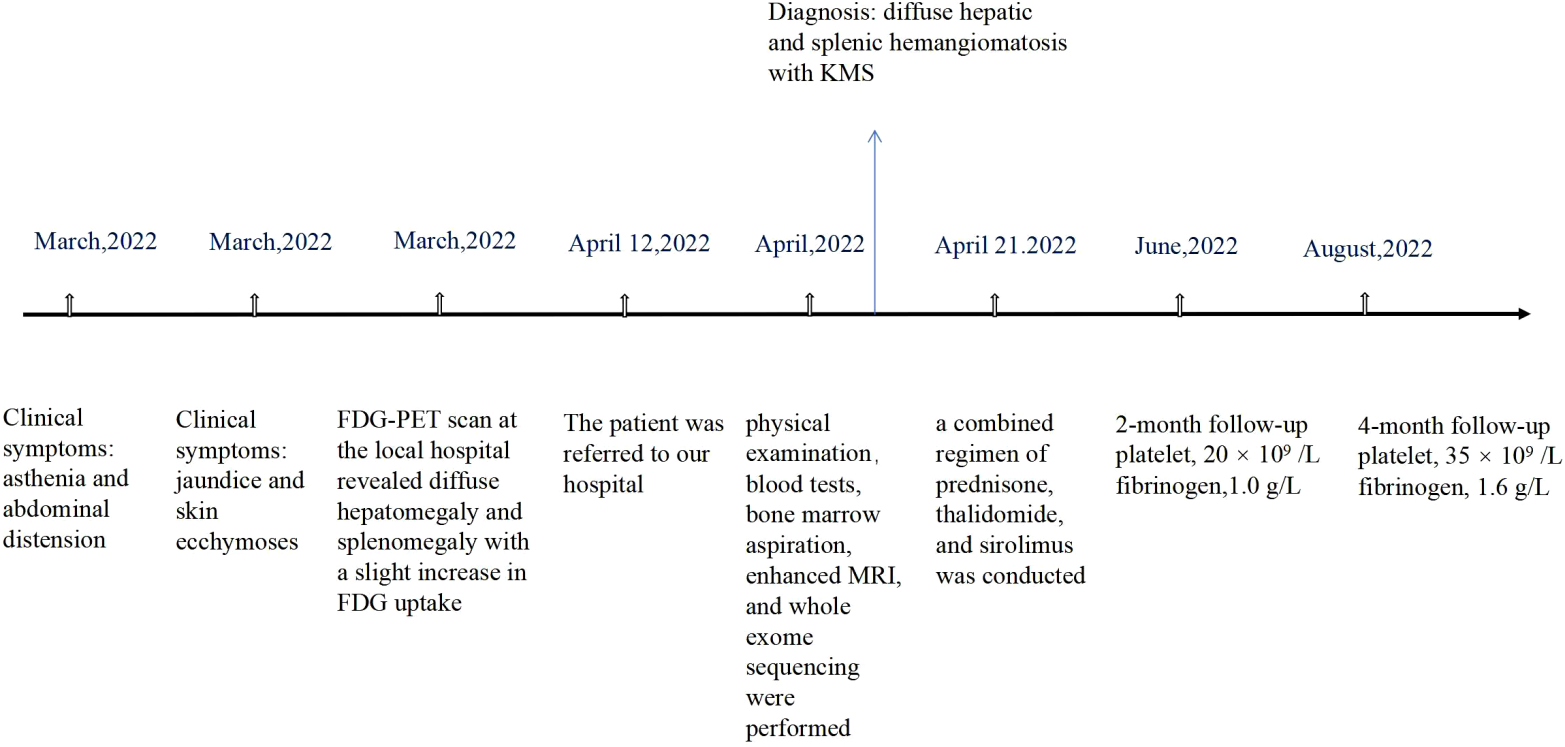
Timeline. FDG-PET, fluorodeoxyglucose positron emission tomography.

Laboratory findings revealed the following abnormalities: White blood cell count, 5.33 (4.0–10.0) × 10^9^/L; hemoglobin, 58 (113–151) g/L; platelet, 9 (101–320) × 10^9^/L; aspartate transaminase, 84 (13–35) U/L; alanine transaminase, 67 (7–40) U/L; total bilirubin, 122.1 (2.0–20.4) μmol/L; direct bilirubin, 61.2 (0–6.8) μmol/L; albumin, 37.2 (40–55) g/L; prothrombin time, >100 (9.0–12.5) s; activated partial thromboplastin time, 35.2 (25.2–38.5) s; fibrinogen, 0.37 (2.0–4.0) g/L; fibrinogen degradation product, 278.4 (0–5) mg/L; international normalized ratio, >8.55 (0.8–1.2); d-dimer, 101.66 (0–0.50) mg/L; α-fetoprotein, 2.0 (<8.1) ng/ml; carbohydrate antigen, 19–9, 9.9 (0–37) U/ml; and carcinoembryonic antigen, 1.0 (0–5.0) ng/ml (**Supplementary Table S1**). Anti-hepatitis B antigen and anti-hepatitis C antibody tests were negative. Hemolytic tests, including glucose 6-phosphate dehydrogenase activity and Coombs, were normal. The autoantibody profiles, including anti-dsDNA, antinuclear, anti-histone, and anti-SSA antibodies, were negative. DNA tests for Epstein–Barr virus and cytomegalovirus were negative. The MRI showed hepatosplenomegaly with numerous variable-sized nodules in the whole liver and spleen, which showed hypointensity on T1-weighted images and hyperintensity on T2-weighted images and inhomogeneous enhancement in diffusion MRI (**Figure 1**). Bone marrow aspiration showed reactive bone marrow proliferation, and peripheral blood smear showed fragments of red blood cells. Due to her poor clinical condition, the patient could not cooperate with the liver biopsy. Finally, the clinical symptoms, laboratory tests, and imaging findings led to a diagnosis of diffuse hepatic and splenic hemangiomatosis with KMS. Peripheral blood samples were used to perform whole exome sequencing (WES, The Beijing Genomics Institute, Shenzhen), and the results showed compound heterozygous variants of the *CTC1* gene with a missense mutation, c.3074C>T (p.Ala1025Val) (exon 19), and a splicing site mutation, c.435+9A>C (IVS3) (**Figure 2**). The pathogenicity of both mutations remains unknown. Embolization and surgery could not be performed because of the extensive lesion. After multidisciplinary discussions, the patient was treated with prednisone, 60 mg/day (reduced gradually after 3 weeks); thalidomide, 100 mg/day; and sirolimus, 1 mg/day. She was discharged 2 days after receiving the medications at her and her family’s request. After discharge, the patient returned to her hometown and continued the medications as prescribed. Due to the coronavirus disease 2019 (COVID-19) epidemic, the patient failed to make regular outpatient visits. Through telephonic follow-up, we found that the patient’s abdominal distension and fatigue were reduced, and the ecchymosis had disappeared after 1 month of treatment, while blood checks were not performed this time. After 2 months of taking the medications, the patient had a platelet count of 20 × 10^9^/L and a fibrinogen level of 1.0 g/L. Her anemia, thrombocytopenia, and coagulation function gradually improved 4 months later. Laboratory tests revealed the following: leukocyte count, 6.8 × 10^9^/L; hemoglobin, 90 g/L; platelets, 35 × 10^9^/L; prothrombin time, 13 s; activated partial thromboplastin time, 30 s; and fibrinogen, 1.6 g/L. Although her condition improved, the patient did not consent to CT/MRI review. No significant treatment-related side effects were observed. Relevant clinical courses are shown in **Figure 3**.

The patient provided written informed consent for her data to be used in this study, which was approved by the ethics committee of our institution in line with the tenets of the Declaration of Helsinki.

## Discussion

Liver hemangiomas, the most common benign liver tumor, can be asymptomatic; however, when the hemangiomas are giant or diffuse, various complications can occur (6). Due to the rarity of adult diffuse hepatic hemangiomatosis, available knowledge is limited and derived mostly from case reports. Diffuse hemangiomatosis occurs in the liver and extrahepatic organs, including the spleen, pancreas, or colon (1, 7). MRI is the most sensitive method for diagnosing hepatic hemangiomas and shows high signal intensity on T2-weighted images with gradual enhancement, which is important for assessing extrahepatic involvement (8). Herein, we report a case of adult diffuse hemangiomatosis of the liver and spleen, which was complicated by severe thrombocytopenia, microangiopathic hemolytic anemia, and consumptive coagulopathy, a lethal condition known as KMS (9, 10).

Hemangiomatosis is a vascular abnormality with an unknown etiology. Etiologic links have been reported between hemangiomatosis and inherited systemic diseases, such as Rendu–Osler–Weber disease and skeletal hemangiomatosis, and between hemangiomatosis and the use of medications, such as metoclopramide and estrogen (11, 12). The correlation between hypothyroidism and diffuse hepatic hemagiomatosis has been described in infants but not in adults (13). In our case, the patient denied any medical history or history of metoclopramide and estrogen intake. Whole exome sequencing was performed to explore the etiology, which revealed compound heterozygous variants of the *CTC1* gene: a splicing site mutation, c.435+9A>C, and a novel missense mutation, c.3074C>T (p.Ala1025Val). The *CTC1* gene facilitates proper telomere replication and is associated with telomere biology disorders; it has been proven to be relevant in two traditional telomeropathies, CRMCC and DC (14). The main clinical manifestations of CRMCC are retinal telangiectasia and exudation, leukoencephalopathy, parenchymal cysts, and intracranial calcification. Our patient had no ophthalmological and neurological manifestations, and the PET-CT did not show remarkable neuroimaging features of CRMCC, which excluded the diagnosis of CRMCC with later onset. The main clinical features of DC are nail dystrophy, leukoplakia, reticular pigmentation, and bone marrow failure (15). Moreover, the pathogenic variation of *CTC1* has been reported to be associated with abnormal manifestations of progeria complicated by recurrent fractures (3). Compared with DC patients, the risk of alimentary tract hemorrhage is higher in patients with CRMCC due to hemangiectasis, which has a higher proportion of *CTC1* gene mutations (16). The specific mechanism of CRMCC-related gastrointestinal bleeding remains unclear. However, endoscopy reveals a high proportion of angiectasis in these patients. A mutation in the *CTC1* gene may be involved in the pathological process of vascular diseases (17). Hemangiomatosis is considered an angiogenesis or malformation, modulated by many factors; whether the *CTC1* gene mutations are involved remains unknown.

Owing to the rarity of the disease, most KMS studies are on neonates and infrequently involve adults. The pathological process of KMS involves the activation and capture of platelets and coagulation factors by the vascular lesions, which causes thrombocytopenia, disseminated intravascular coagulation, and even massive hemorrhage. Vascular lesions correlated with KMS include giant vascular tumors, vascular malformations, and vascular fistulas (18). Furthermore, immune activation, cytokines, and inflammation are considered important contributors to promoting the progression of KMS (19).

The treatment of KMS is challenging. The main goal of therapy is to relieve symptoms and rapidly reduce bleeding. As platelet transfusion can worsen the condition, it is only recommended in emergencies. Surgical management, endovascular intervention, and radiation have been proven to be effective, especially for KMS resulting from a single lesion (20). For diffuse hemangiomatosis with KMS, radiotherapy, endovascular interventional embolization, and other medications did not show significant efficacy. Liver transplantation was considered a radical treatment for diffuse hepatic hemangioma and did not apply to cases with extrahepatic lesions (1). To date, no standard therapeutic guidelines are available for KMS; several therapies, such as vincristine, glucocorticoids, thalidomide, sirolimus, and antivascular endothelial growth factor agents, have been applied in the treatment of KMS (21). Ultimately, our patient received a combination regimen of prednisone, sirolimus, and thalidomide, which led to an improvement. Compared with KMS resulting from a single lesion or cutaneous hemangioma, the response to medications in diffuse hemangiomatosis with KMS is worse (12). The improvement of our patient’s platelet count and coagulation function did not reach the expected level after receiving combined therapy, which may be due to diffuse liver infiltration and extrahepatic lesions. For diffuse hemangiomatosis with KMS, more clinical studies are required to explore the best therapeutic regimen.

In conclusion, we report a case of diffuse hemangiomatosis in the liver and spleen complicated by KMS in a 59-year-old woman who carried two novel heterozygous *CTC1* gene variants. Further investigation is required to determine the link between the *CTC1* gene mutations and hemangiomatosis. For KMS associated with diffuse hemangiomatosis, which is considered inoperable, a combined regimen of prednisone, sirolimus, and thalidomide can improve survival.

## Patient perspective

I began to feel abdominal distension and fatigue, which gradually worsened; it was difficult for me to even eat. I received blood and FDG-PET examinations at a nearby hospital, but the diagnosis was still not precise. After blood transfusions and other treatments, my symptoms became worse. Later, I visited this hospital with my daughter. After careful examinations and multidisciplinary discussions, they gave me a treatment plan. After understanding the severity and rarity of my condition, I hoped to spend more time with my family. I returned to my hometown and continued the medications as prescribed. Over the past few months, my condition has gradually improved.

## Data availability statement

The datasets presented in this study can be found in online repositories. The names of the repository/repositories and accession number(s) can be found in the article/**Supplementary Material**.

## Ethics statement

Written informed consent was obtained from the individual(s) for the publication of any potentially identifiable images or data included in this article.

## Author contributions

XH wrote the manuscript. ZW,G and XM,N reviewed and edited the manuscript. All authors contributed to the article and approved the submitted version.

## Supplementary material

Supplementary Table 1 Laboratory data on admission.

## References

[1] Shimizu Y Komura T Seike T Omura H Kumai T Kagaya T. A case of an elderly female with diffuse hepatic hemangiomatosis complicated with multiple organic dysfunction and kasabach-Merritt syndrome. Clin J Gastroenterol (2018) 11:411–6. doi: 10.1007/s12328-018-0871-3 29845554

[2] Zhang XM Tong Y Li Q He Q. Diffused hepatic angiosarcoma with kasabach-Merritt syndrome-case report and literature review. BMC Gastroenterol (2020) 20:80. doi: 10.1186/s12876-020-01216-z 32228471 PMC7104501

[3] Sargolzaeiaval F Zhang J Schleit J Lessel D Kubisch C Precioso DR. CTC1 mutations in a Brazilian family with progeroid features and recurrent bone fractures. Mol Genet Genomic Med (2018) 6:1148–56. doi: 10.1002/mgg3.495 PMC6305643 30393977

[4] Kim B Yun W Lee ST Choi JR Yoo KH Koo HH. Prevalence and clinical implications of germline predisposition gene mutations in patients with acute myeloid leukemia. Sci Rep (2020) 10:14297. doi: 10.1038/s41598-020-71386-z 32868804 PMC7459095

[5] Romaniello R Arrigoni F Citterio A Tonelli A Sforzini C Rizzari C. Cerebroretinal microangiopathy with calcifications and cysts associated with CTC1 and NDP mutations. J Child Neurol (2013) 28:1702–8. doi: 10.1177/0883073812467849 23220793

[6] Zhao Y Legan CE. Liver transplantation for giant hemangioma complicated by kasabach-Merritt syndrome: A case report and literature review. Am J Case Rep (2022) 23:e936042. doi: 10.12659/AJCR.936042 35796001 PMC9136188

[7] Zhang WH Luo FQ Wang H. Diffuse infiltrative hemangioma of pancreas accompanied by kasabach-Merritt syndrome: A case report. Chin Med J (Engl). (2020) 133:2263–5. doi: 10.1097/CM9.0000000000001037 PMC7508427 32826612

[8] González-Nieto MI Escobar HL. A case of diffuse hepatic hemangiomatosis coexistent with giant hemangioma: Case report and literature review. Radiol Case Rep (2021) 16:1518–23. doi: 10.1016/j.radcr.2021.03.058 PMC8052474 33903807

[9] Ji Y Chen S Yang K Xia C Li L. Kaposiform hemangioendothelioma: Current knowledge and future perspectives. Orphanet J Rare Dis (2020) 15:39. doi: 10.1186/s13023-020-1320-1 32014025 PMC6998257

[10] Lewis D Vaidya R. Kasabach Merritt Syndrome. StatPearls [Internet]. Treasure Island (FL): StatPearls Publishing (2022). 30085595

[11] Ota T Kamiyama T Kato T Hanamoto T Hirose K Otsuka N. A rare case of cavernous hemangioma accompanied with diffuse hepatic hemangiomatosis. Surg Case Rep (2020) 6:251. doi: 10.1186/s40792-020-01023-4 33001265 PMC7530161

[12] He S Chen W Yang Y Tang X Zhou G Zhou J. Adult diffuse hepatic hemangiomatosis: A case report and review of the literature. Clin Res Hepatol Gastroenterol (2022) 46:101789. doi: 10.1016/j.clinre.2021.101789 34384928

[13] Bhardwaj N Parkhi M Kumar M Kaman L Mitra S. Adult diffuse hepatic hemangiomatosis. Autops Case Rep (2022) 12:e2021401. doi: 10.4322/acr.2021.401 36186112 PMC9524384

[14] Grill S Nandakumar J. Molecular mechanisms of telomere biology disorders. J Biol Chem (2021) 296:100064. doi: 10.1074/jbc.REV120.014017 33482595 PMC7948428

[15] Lyu X Sang PB Chai W. CST in maintaining genome stability: Beyond telomeres. DNA Repair (Amst). (2021) 102:103104. doi: 10.1016/j.dnarep.2021.103104 33780718 PMC8081025

[16] Niewisch MR Savage SA. An update on the biology and management of dyskeratosis congenita and related telomere biology disorders. Expert Rev Hematol (2019) 12:1037–52. doi: 10.1080/17474086.2019.1662720 PMC9400112 31478401

[17] Himes RW Chiou EH Queliza K Shouval DS Somech R Agarwal S. Gastrointestinal hemorrhage: A manifestation of the telomere biology disorders. J Pediatr (2021) 230:55–61. doi: 10.1016/j.jpeds.2020.09.038 32971146

[18] Famularo G Galluzzo M Ottaviani P Tarsitani P. Kasabach-Merritt syndrome arising from a vascular fistula. Am J Emerg Med (2019) 37:1393–4. doi: 10.1016/j.ajem.2019.04.013 31003830

[19] Borst AJ Nakano TA. Targeting inflammation-induced kasabach-Merritt phenomenon. Blood (2022) 139:1603–5. doi: 10.1182/blood.2022015412 35298606

[20] Yang Y Guo Z Wang Z Luo L Chen Y. Successful management of a pregnant woman with kasabach–Merritt syndrome and preeclampsia. Medicine (2020) 99:e21198. doi: 10.1097/MD.0000000000021198 32664166 PMC7360294

[21] Huang Y Zhou D Han B Li T Wang S. Successful treatment of an adult with kasabach-Merritt syndrome using thalidomide, vincristine, and prednisone. J Int Med Res (2019) 47:1810–4. doi: 10.1177/0300060519830242 PMC6460612 30806107

