# Supplementary material for: Case Report: CTC1 mutations in a patient with diffuse hepatic and splenic hemangiomatosis complicated by Kasabach–Merritt syndrome
Source: Front Oncol. 2023 Jan 25;13:1087790. doi: 10.3389/fonc.2023.1087790 (PMC9905704; doi:10.3389/fonc.2023.1087790)
Supplement: Supplementary Table 1 — Laboratory data on admission. [file Table_1.docx]

**Supplementary Table 1. Laboratory data on admission**

| WBC 5.33× 10^9^ /L | ALT 67U/L | BUN 10.21 mmol/L |
| --- | --- | --- |
| Neutro 69.2% | AST 84 U/L | PT ＞100 s |
| Lymph 20.5% | ALP 231 U/L | APTT 35.2 s |
| Mono 7.3% | LDH 650 U/L | FIB 0.37 g/L |
| Eosino 2.6% | T-BIL 122.1 µmol/L | D-dimer 101.66 mg/L |
| Baso 0.4% | D-BIL 61.2 µmol/L | FDP 278.4 mg/L |
| RBC 2.1× 10^12^/L | I-BIL 60.9 µmol/L |  |
| Hb 58 g/L | Alb 37.2 g/L |  |
| PLT 9 ×10^9^/L | Cr 72 µmol/L |  |
